# Establishment and Validation of a Predictive Model for Radiation-Associated Aspiration Pneumonia in Patients with Radiation-Induced Dysphagia after Nasopharyngeal Carcinoma

**DOI:** 10.1155/2022/6307804

**Published:** 2022-08-19

**Authors:** Honghong Li, Yong He, Xiaohuang Zhuo, Zongwei Yue, Xiaoming Rong, Yike Li, Yi Li, Lei He, Jinping Cheng, Dong Pan, Ruiqi Xue, Jinhua Cai, Jingru Jiang, Yongteng Xu, Yamei Tang

**Affiliations:** ^1^Department of Neurology, Sun Yat-Sen Memorial Hospital, Sun Yat-Sen University, 107 Yan Jiang Xi Road, Guangzhou, China; ^2^Radiotherapeutic Department, The Second Affiliated Hospital of Guangzhou Medical University, Guangzhou, China; ^3^Department of Otolaryngology, Vanderbilt University Medical Center, Nashville, TN, USA; ^4^Guangdong Provincial Key Laboratory of Malignant Tumor Epigenetics and Gene Regulation, Sun Yat-Sen Memorial Hospital, Sun Yat-Sen University, Guangzhou, China; ^5^Guangdong Province Key Laboratory of Brain Function and Disease, Zhongshan School of Medicine, Sun Yat-Sen University, 74 Zhongshan 2nd Road, Guangzhou, China

## Abstract

**Introduction:**

Radiotherapy for patients with head and neck cancers raises their risk of aspiration pneumonia-related death. We aimed to develop and validate a model to predict radiation-associated aspiration pneumonia (RAP) among patients with dysphagia after radiotherapy for nasopharyngeal carcinoma (NPC).

**Materials and Methods:**

A total of 453 dysphagic patients with NPC were retrospectively recruited from Sun Yat-Sen Memorial Hospital from January 2012 to January 2018. Patients were randomly divided into training cohort (*n* = 302) and internal validation cohort (*n* = 151) at a ratio of 2 : 1. The concordance index (C-index) and calibration curve were used to evaluate the accuracy and discriminative ability of this model. Moreover, decision curve analysis was performed to evaluate the net clinical benefit. The results were externally validated in 203 dysphagic patients from the First People's Hospital of Foshan.

**Results:**

Derived from multivariable analysis of the training cohort, four independent factors were introduced to predict RAP, including Kubota water drinking test grades, the maximum radiation dose of lymph node gross tumor volume (Dmax of the GTVnd), neutrophil count, and erythrocyte sedimentation rate (ESR). The nomogram showed favorable calibration and discrimination regarding the training cohort, with a C-index of 0.749 (95% confidence interval (CI), 0.681 to 0.817), which was confirmed by the internal validation cohort (C-index 0.743; 95% CI, 0.669 to 0.818) and the external validation cohort (C-index 0.722; 95% CI, 0.606 to 0.838).

**Conclusions:**

Our study established and validated a simple nomogram for RAP among patients with dysphagia after radiotherapy for NPC.

## 1. Introduction

Radiotherapy-induced dysphagia, with an incidence of 5.7-37.3% in nasopharyngeal carcinoma (NPC) patients [1], usually results in severe pneumonia [2, 3]. Deteriorating swallowing function indicates an increased risk of radiotherapy-associated aspiration pneumonia (RAP) [4], almost twice as high as that of nondysphagia patients [5]. The burden brought by RAP could be tremendous. Not only does it prolong hospital stays and ventilatory support in ICU but severely affects patients' quality of life and even be life-threating [6]. Chen et al. reported 43.9% (18/41) patients needed ventilatory support in ICU and 17.1% (7/41) died for aspiration pneumonia postradiotherapy [7]. In addition, head and neck cancer patients are at a higher risk of RAP-related death [8]. RAP accounts for 34.6% of noncancer-related deaths in patients with NPC, increasing financial burden on the medical system [9]. However, RAP dose not garner sufficient attention. There are some available treatments for dysphagic patients to prevent RAP, such as nasogastric feeding tube and gastrostomy. The limitation is also obvious, as nasogastric feeding tube is only valid for 1 month, and it is costly and inconvenient to replace gastric tube, while gastrostomy is an invasive treatment. Thus, early screening patients with high risk and follow ups could help preventive approach implement, minimize occurrence of RAP, and provide precautions.

Nomogram serves as a reliable tool to quantify risk for various diseases [10]. However, nomogram for predicting RAP after radiotherapy in dysphagic patients was yet to be developed. In this study, we sought to develop a useful and practical nomogram for prediction of RAP by combining common clinical variables, aiming to aid clinical decision making and improve curative effect. Additionally, we externally validate the model using a separate cohort from the First People's Hospital of Foshan.

## 2. Materials and Methods

### 2.1. Patient Characteristics

In this study, we reviewed the charts of patients who were diagnosed with radiotherapy-induced dysphagia after radiotherapy for NPC between January 2012 and January 2018 in Sun Yat-Sen Memorial hospital. The diagnostic criteria of dysphagia were referred to previously studies using video fluoroscopy swallowing study (VFSS) [11]. Eligibility criteria for inclusion were described as follows: (a) age ≥ 18 years, (b) history of radiotherapy for NPC, and (c) evidence of dysphagia. Excluded criteria were as follows: (a) with NPC metastasis involving the low cranial nerves leading to dysphagia, (b) with evidence of dysphagia unrelated to radiotherapy, and (c) suffering from pneumonia. The eligible patients from Sun Yat-Sen Memorial hospital were randomly classified into training cohort and internal validation cohort. To examine the generalizability of the model, we used data from patients at the First People's Hospital of Foshan between January 2012 and January 2018 as an external validation cohort with the same inclusion and exclusion criteria.

### 2.2. Date Collection

Age, sex, current smoking, diet way, outcome of Kubota water drinking test, whether treated with steroid, occurrence of RAP, blood routine, low-density lipoprotein (LDL), prealbumin, albumin, high-sensitivity c-reactive protein (hsCRP), and erythrocyte sedimentation rate (ESR) were attained from medical records. The TNM stage, radiotherapy technique (conventional radiotherapy) or intensity-modulated radiotherapy (IMRT), chemotherapy, the maximum radiation dose of nasopharynx gross tumor volume (*D*_max_ of the GTVnx), and the maximum radiation dose of lymph node gross tumor volume (*D*_max_ of the GTVnd) were also recorded. All patients were restaged based on the 8th American Joint Committee on Cancer Union for International Cancer Control TNM staging manual [12]. The primary endpoint was the occurrence of RAP.

RAP was defined as the pneumonia caused by inhalation of food or vomitus [13], which was confirmed by definite findings in chest X-ray and findings of pneumonia in radiographs according to previous study [14]. The RAP was diagnosed with the following criteria [15]: (a) patients had both clinical manifestations, laboratory, and radiographic evidence of pneumonia and (b) no evidence of pneumonia caused by microorganisms.

### 2.3. Construction of the Nomogram

The construction of the nomogram was performed in training cohort in R-environment. First, we used univariate Cox proportional hazard to reduce candidate predictors according to *P* < 0.05. Second, the independent predictors of RAP were defined by the multivariate Cox proportional hazard regression model with backward-selection procedure using Akaike's Information Criterion [16]. Thus, the nomogram was formulated by the R package of *rms* according to the result of multivariate analysis.

### 2.4. Validation and Calibration of the Nomogram

The internal validation and external validation of the nomogram were performed by 1000 bootstrap resamples. The concordance index (C-index) was used to evaluate the discrimination ability of the nomogram. The values of the C-index ranged from 0.5 to 1.0, which means a random chance when it is 0.5, and perfect discriminate ability when it is 1 [17]. Calibration for the 1-year and 3-year pneumonia-free survival (PFS) was performed via comparing the predicted survival with the observed survival after bias correction.

### 2.5. Risk Group Stratification and Clinical Usefulness of the Nomogram

Risk scores of every patient can be calculated based on the established nomogram. The maximally selected rank statistics as implemented in the “*maxstat*” R package was conducted to stratified patients into high-risk and low-risk of RAP with a cutoff value of 12.3. Then, the cutoff value was applied to these two validation cohorts. Kaplan-Meier survival curves were formed compared with the log-rank test. To evaluate the clinical usefulness of this predictive model, the decision curve analysis (DCA) was performed through calculating the net benefits for a range of threshold probabilities among these three cohorts [18]. DCA was used to assess whether the decisions based on the current nomogram could improve patient's outcome.

### 2.6. Statistical Analyses

Continuous variables were converted to categorical variables according to the median number [19]. Proportional hazard assumption was verified by the Schönfeld test. All statistical tests were performed using the R for Windows (version 3.4.2, http://www.r-project.org/). The Cox proportional hazards regression model analysis was conducted using the “*survival*” package and “*MASS*” package. The “*rms*” package was used to perform the nomogram and calibrations plots. The function “*stdca. R.*” was used to perform the DCA. All statistical tests were two-tailed, and *P* value < 0.05 was considered significant.

## 3. Results

### 3.1. Clinical Characteristics

A total of 453 dysphagic patients with NPC were recruited from Sun Yat-Sen Memorial hospital. Randomly divided into two sets at a ratio of 2 : 1, 302 patients were assigned to training cohort and 151 patients to internal validation cohort. The external validation cohort consisted of 203 patients with dysphagia from the First People's Hospital of Foshan. The screening process was shown in Supplementary Figure 1, and the clinical characteristics of patients were listed in Table 1. The median follow-up time was 2.5 years (interquartile range (IQR) 0.7-5.4) for the training cohort, 2.5 years (IQR 1.0-6.0) for the internal validation, and 2.3 years (IQR 1.2-4.4) for the external validation cohort. The ratio of patients suffering from RAP at least once in the next 3 years after diagnosis of dysphagia was 19.5% (59/302), 21.2% (32/151), and 15.3% (31/203) in the training, internal validation, and external validation cohorts, respectively. Regarding the incidence of RAP, there was no significant difference among three groups (*P* = 0.312).

### 3.2. Predictors for RAP and Nomogram Construction

**Figure 1:**
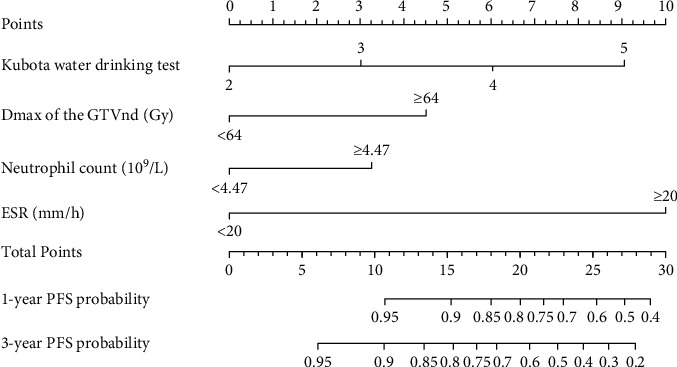
Nomogram to predict 1- and 3-year PFS rates. Points were assigned for Kubota water drinking test, *D*_max_ of the GTVnd, neutrophil count, and ESR level by drawing a line upward from the corresponding values to the “Points” line. The sum of these four points, plotted on the “Total points” line corresponds to predictions of 1- and 3-year PFS. Abbreviations: PFS: pneumonia-free survival; *D*_max_ of the GTVnd: the maximum radiation dose of lymph node gross tumor volume; ESR: erythrocyte sedimentation rate; RAP: radiation-associated aspiration pneumonia.

The univariate Cox regression analysis revealed 12 clinical variable candidates in the training cohort (*P* < 0.05), which included diet, Kubota water drinking test grade, steroid usuage, *D*_max_ of the GTVnd, traditional radiotherapy, neutrophil count, blood Hb, LDL, prealbumin and albumin, hsCRP, and ESR. Among these factors, 4 significant predictors (including Kubota water drinking test grade, *D*_max_ of the GTVnd, neutrophil count, and ESR) were identified as independent factors by multivariate Cox proportional hazards regression model (Table 2). The pH assumption was met as the Schönfeld test demonstrated (Supplementary Figure 2, *P* = 0.6889). Then, the nomogram was established based on the overall consideration of these four factors (Figure 1). The nomogram indicated that swallowing function and ESR had major contribution to RAP occurrence, followed by *D*_max_ of the GTVnd and neutrophil count (Figure 1). Each factor of these variables had a corresponding score on the point scale. It was accessible to estimate the probability of RAP based on the total score with a clear boundary.

### 3.3. Calibration and Validation of the Nomogram

**Figure 2:**
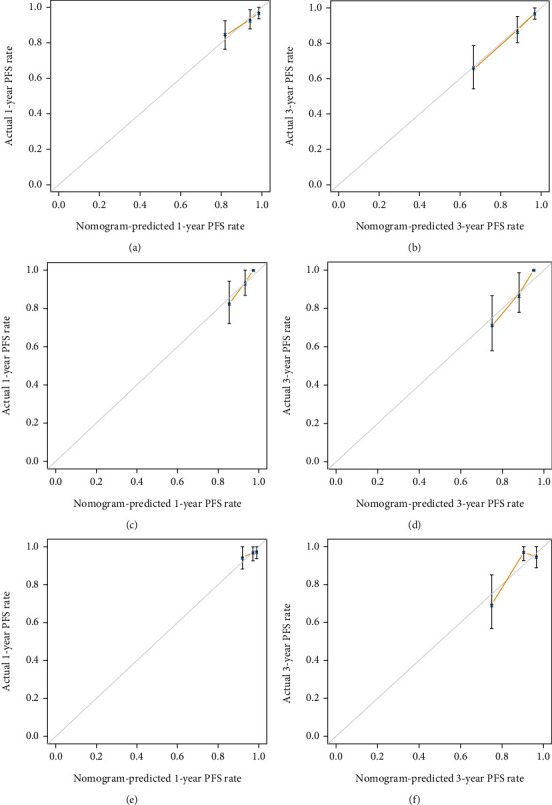
Calibration curves of the nomogram. The observed PFS is shown compared with the nomogram at (a) 1 year and (b) 3 years in the training cohort, at (c) 1 year and (d) 3 years in the internal validation cohort, and at (e) 1 year and (f) 3 years in the external validation cohort. The calibration curves depict the calibration of the nomogram in terms of the agreement between the predicted risk of RAP and the observed RAP outcomes. The 45-degree gray line represents a perfect prediction, and the yellow solid lines represent the predictive performance of the nomogram. The distance between the yellow solid line and the ideal line represents the superior predictive accuracy of the nomogram. Abbreviations: PFS: pneumonia-free survival; RAP: radiation-associated aspiration pneumonia.

In the training cohort, the C-index was 0.749 (95% confidence interval (CI), 0.681 to 0.817) suggesting a favorable discrimination. The calibration curves for the RAP rate at 1 year and 3 years indicated favorable agreement between the predict model and actual observation (Figures 2(a) and 2(b)). The satisfactory calculation of the nomogram was confirmed using the internal validation cohort (Figures 2(c) and 2(d)) and external validation cohort (Figures 2(e) and 2(f)). Moreover, the C-index was 0.743 (95% CI, 0.669 to 0.818) and 0.722 (95% CI, 0.606 to 0.838) in internal and external validation cohort, respectively, both with a good discrimination.

### 3.4. Performance of the Nomogram in Stratifying Risk of Patients and Its Clinical Implication

**Figure 3:**
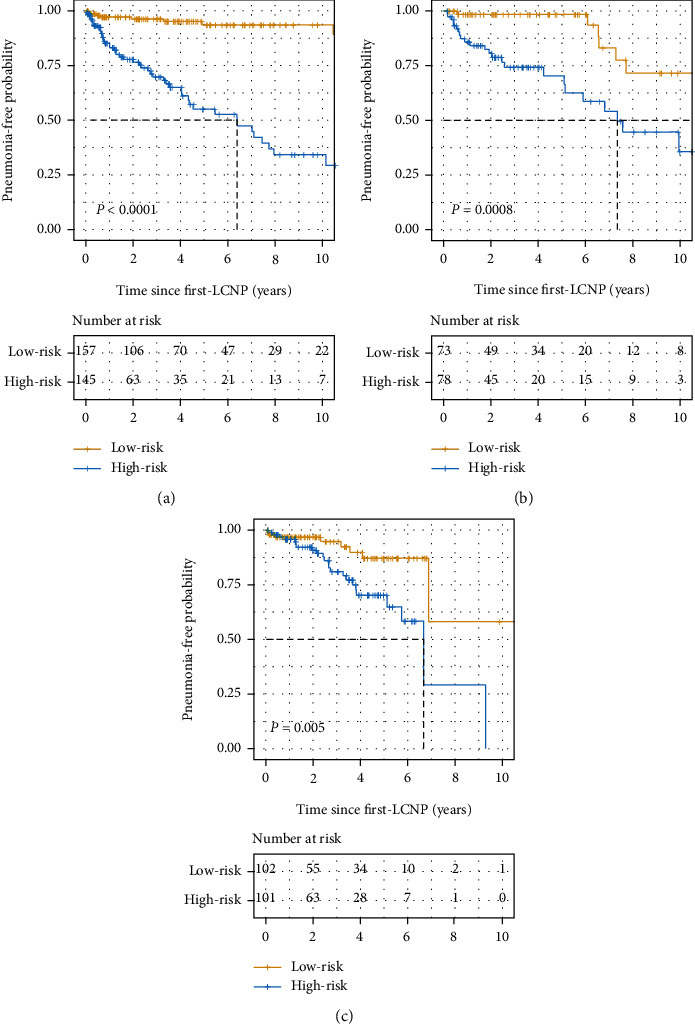
Kaplan–Meier survival curves of the cohorts categorized into low- and high-risk groups. A significant association between the risk score and PFS was observed using the training cohort (a) and confirmed using the internal (b) and external validation cohorts (c). Abbreviations: PFS: pneumonia-free survival.

**Figure 4:**
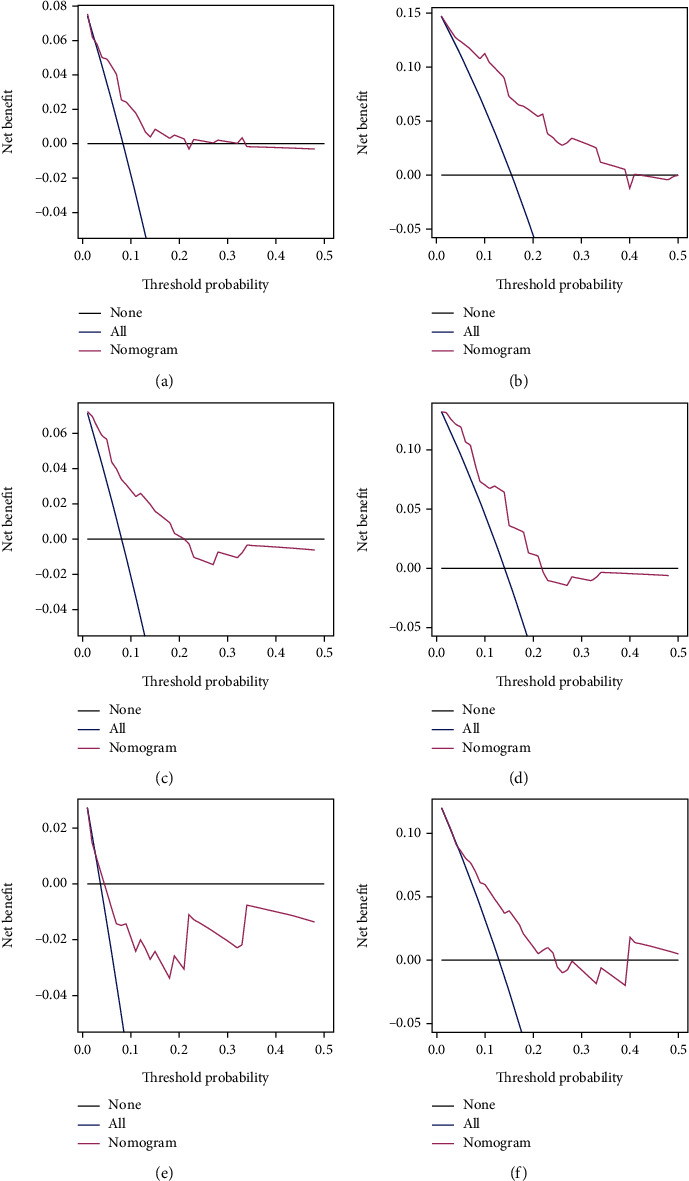
DCA of the nomogram. Decision curves for PFS at (a) 1 year and (b) 3 years in the training cohort, at (c) 1 year and (d) 3 years in the internal validation cohort, and at (e) 1 year and (f) 3 years in the external validation cohort were applied to the nomogram. The *x*-axis represents the threshold probability. The *y*-axis measures the net benefit. The black line depicts the net benefit of the strategy of treating no patients. The blue line depicts the net benefit of the strategy of treating all patients. The red line represents the nomogram. The net benefit was calculated by subtracting the proportion of all patients who are false positive from the proportion who are true positive, weighting by the relative harm of forgoing treatment compared with the negative consequences of an unnecessary treatment. The threshold probability is where the expected benefit of treatment is equal to the expected benefit of avoiding treatment. For example, if the possibility of RAP development in a patient is over the threshold probability, then, a RAP treatment strategy should be adopted. Abbreviations: DCA: decision curve analysis; PFS: pneumonia-free survival; RAP: radiation-induced aspiration pneumonia.

By applying our nomogram, the patients were divided into low-risk and high-risk groups with a cutoff value of 12.3. Satisfactory discrimination between RAP of the high-risk and low-risk patients was observed in the training cohort (Figure 3(a), *P* < 0.0001), also confirmed by both internal (Figure 3(b), *P* = 0.0008) and external validation cohort (Figure 3(c), *P* = 0.005). Therefore, our nomogram served well in identifying the high-risk RAP patients after diagnosis of dysphagia. DCA was formed to estimate the usefulness of the model in a clinical context as shown in Figure 4. These plots suggested that this nomogram could guide clinical decisions and improve therapeutic effect compared to nonselective treatment or nontreated for a risk probability ranging between 0.03 to 0.21 and 0.02 to 0.39 for 1-year and 3-year predictions in training cohort (Figures 4(a) and 4(b)). The usefulness of this nomogram to predict the 1-year PFS in external cohort is not satisfactory (Figure 4(e)).

## 4. Discussion

We established and validated an effective and useful predictive nomogram model to identify patients at high risk of RAP among those with dysphagia after radiotherapy for NPC. This novel prediction instrument was successfully internally and externally validated in separate cohorts and showed good discrimination and calibration. The model incorporated four factors that should be evaluated with priority in clinical practice, including Kubota water drinking test grades, *D*_max_ of the GTVnd, neutrophil count, and ESR. Our data indicated that a higher Kubota water drinking test grade predicted for a higher risk of RAP. Kubota water drinking test, a simple and routine test for dysphagic patients, is used to assess the severity of dysphagia, and the higher grade indicates more serious swallowing dysfunction [11]. In line with these findings, there is emerging evidence that patients with poor swallowing function were more likely to develop RAP [20, 21], and careful oral management for swallowing can reduce the incidence of RAP [22, 23]. Moreover, aspiration and dysphagia after radiotherapy are regarded as the main cause of RAP in NPC patients. Identification of early swallowing dysfunction with Kubota water drinking test could screen out patients at a high-risk of RAP and facilitate targeted follow up and clinical decision making to minimize the risk of RAP.

Increased *D*_max_ of the GTVnd was found to increase the risk of RAP in our patients. Previous researches have found a close relationship between RAP and increased radiation dose [24, 25]. *D*_max_ of the GTVnd refers to the maximum radiation dose of neck lymph node gross tumor volume where the low cranial nerves lie. In addition, neck radiotherapy leads to dysphagia by damaging the neck fat, fascia, and neuron axons [26]. All the above might be the underlying mechanism for the association of increased *D*_max_ of the GTVnd with risk of RAP.

Remarkably, our study found that the neutrophil count also impacted on risk of RAP. After radiotherapy, tumor cells could recruit inflammatory cells from bone-marrow, including macrophages and neutrophil [27]. Several studies have suggested that higher circulating neutrophil count indicated worse overall survival outcomes in different tumor models in patients undergoing radiotherapy [28, 29]. High levels of neutrophil reflected the systemic inflammatory state [30]. Patients with high levels of neutrophil count were more susceptible to infection. Our results also found that ESR was an independent risk factor for RAP. ESR is nonspecific but one of the most commonly used laboratory markers for systemic inflammatory response in clinical practice [31]. ESR might promote RAP through enhancing postradiation inflammatory reaction.

Our study possessed several strengths. First, our nomogram was established through analysis of easily measured and routinely available predictors in a well-characterized training cohort of dysphagic patients. Second, our model has been successfully validated in two independent cohorts, thereby improving the generalizability as well as credibility. Both physicians and patients could utilize this easy-to-use model to assess the risk of RAP after the occurrence of dysphagia. Identifying patients at high risk for RAP benefits personalized treatment. However, as for those patients with dysphagia who need additional rehabilitation exercise and close follow-up, the intervention remains controversial [32]. This model would have tremendous help to address such issues. Patients at high risk of RAP should be closely followed up, reduce risk factors of RAP, enhance nutrient intake, and seek lifestyle guidance. Additionally, our model might provide information for RAP risk stratification in clinical study.

Nevertheless, there are still several limitations of the study. First, our study was a retrospective study, and only patients with dysphagia following irradiation for NPC were included. Further studies are warranted to explore whether nomogram can be extended to patients undergoing radiotherapy for other types of tumors. Second, the clinical factors selected as potential predictors for RAP were based on previously studies and our clinical experiences. Some unrecorded clinical factors including rehabilitation exercise, number of irradiation fields, and field arrangement might also be associated. Additionally, this nomogram model was developed from a Chinese people cohort. The usefulness of this nomogram to predict the 1-year PFS in external cohort is not satisfactory. Thus, a multicenter prospective study with different ethnicities may deserve further confirmation.

## 5. Conclusion

In conclusion, we have established and validated a predictive model, which could help identify patients at a high risk of developing RAP. Using this nomogram, physicians could more precisely evaluate the incidence of RAP among dysphagic patients after radiotherapy and identify high-risk RAP patients who require long-term individualized treatment. However, further prospective training and validation of the model are warranted to confirm a reliable tool to predict RAP.

## Figures and Tables

**Table 1:** Demographics of dysphagic patients after radiotherapy for NPC.

Characteristic	Training cohort (*N* = 302)	Internal validation cohort (*N* = 151)	External validation cohort (*N* = 203)	*P*
Age, years (<51 vs. ≥51)	149 (49.3) vs. 153 (50.7)	71 (47.0) vs. 80 (53.0)	73 (36.0) vs. 130 (64.0)	0.010
Sex (male vs. female)	232 (76.8) vs. 70 (23.2)	113 (74.8) vs. 38 (25.2)	140 (69.0) vs. 63 (31.0)	0.137
Current smoking (no vs. yes)	280 (92.7) vs. 22 (7.3)	128 (84.8) vs. 23 (15.2)	NA vs. NA	0.008
Diet				0.864
Oral diet	282 (93.4)	140 (92.7)	NA	
Gastrostomy	9 (3.0)	4 (2.7)	NA	
Nasogastric	11 (3.6)	7 (4.6)	NA	
Kubota water drinking test				<0.001
Grade 2	188 (62.2)	93 (61.6)	48 (23.6)	
Grade 3	50 (16.6)	21 (13.9)	53 (26.1)	
Grade 4	55 (18.2)	29 (19.2)	95 (46.8)	
Grade 5	9 (3.0)	8 (5.3)	7 (3.4)	
Steroid (no vs. yes)	111 (36.8) vs. 191 (63.2)	49 (32.5) vs. 102 (67.5)	143 (70.4) vs. 60 (29.6)	<0.001
*D*_max_ of the GTVnx, Gy (<70 vs*.National Natural Science Foundation of China*≥70)	79 (26.2) vs. 223 (73.8)	27 (17.9) vs. 124 (82.1)	79 (39.0) vs. 124 (61.0)	<0.001
*D*_max_ of the GTVnd, Gy (<64 vs. ≥64)	165 (54.6) vs. 137 (45.4)	72 (47.7) vs. 79 (52.3)	110 (54.2) vs. 93 (45.8)	0.341
Radiotherapy methods (conventional vs. IMRT)	125 (41.4) vs. 177 (58.6)	97 (64.2) vs. 54 (35.8)	85 (41.9) vs. 118 (58.1)	<0.001
Neutrophil count, ×10^9^/L (<4.47 vs. ≥4.47)	150 (49.7) vs. 152 (50.3)	77 (51.0) vs. 74 (49.0)	91 (44.8) vs. 112 (55.2)	0.441
Hemoglobin, g/L (<127.5 vs. ≥127.5)	151 (50.0) vs. 151 (50.0)	85 (56.3) vs. 66 (43.7)	NA vs. NA	0.206
LDL, mmol/L (<3.25 vs. ≥3.25)	147 (48.7) vs. 155 (51.3)	86 (57.0) vs. 65 (43.0)	96 (47.3) vs. 107 (52.7)	0.155
Prealbumin, mg/L (<0.24 vs. ≥0.24)	149 (49.3) vs. 153 (50.7)	87 (57.6) vs. 64 (42.4)	NA vs. NA	0.096
Albumin, g/L (<39.3 vs. ≥39.3)	150 (49.7) vs. 152 (50.3)	76 (50.3) vs. 75 (49.7)	57 (28.1) vs. 146 (71.9)	<0.001
hsCRP, mg/L (<4.70 vs. ≥4.70)	151 (50.0) vs. 151 (50.0)	72 (47.7) vs. 79 (52.3)	140 (69.0) vs. 63 (31.0)	<0.001
ESR, mm/h (<20 vs. ≥20)	145 (48.0) vs. 157 (52.0)	68 (45.0) vs. 83 (55.0)	115 (56.7) vs. 88 (43.3)	0.062
TNM stage				<0.001
Stage 1	8 (2.6)	4 (2.6)	2 (1.0)	
Stage 2	35 (11.6)	34 (22.5)	17 (8.4)	
Stage 3	162 (53.7)	54 (35.8)	64 (31.5)	
Stage 4	97 (32.1)	59 (39.1)	120 (59.1)	
Secondary radiotherapy (no vs. yes)	273 (90.4) vs. 29 (9.6)	130 (86.1) vs. 21 (13.9)	NA vs. NA	0.168
Chemotherapy (no vs. yes)	243 (80.5) vs. 59 (19.5)	118 (78.1) vs. 33 (21.9)	NA vs. NA	0.563
Median follow-up duration (IQR; years)	2.5 (0.7-5.4)	2.5 (1.0-6.0)	2.3 (1.2-4.4)	0.381
Pneumonia (no vs. yes)	243 (80.5) vs. 59 (19.5)	119 (78.8) vs. 32 (21.2)	172 (84.7) vs. 31 (15.3)	0.312

Data are shown as numbers (%) or medians (interquartile ranges).

**Table 2:** Risk factors for radiation-associated aspiration pneumonia.

Variable	Univariate cox regression		Multivariate cox regression	
	HR (95% CI)	*P*	HR (95% CI)	*P*
Age, years (<51 vs. ≥51)	1.458 (0.856-2.485)	0.166		
Sex (male vs. female)	0.824 (0.437-1.553)	0.549		
Current smoking (no vs. yes)	1.165 (0.466-2.916)	0.744		
Diet		0.029		
Oral diet	Reference			
Gastrostomy	0.758 (0.105-5.497)	0.784		
Nasogastric tube	3.448 (1.370-8.677)	0.009		
Kubota water drinking test		<0.001		0.002
Grade 2	Reference		Reference	
Grade 3	1.553 (0.698-3.454)	0.280	1.311 (0.583-2.946)	0.513
Grade 4	3.609 (2.038-6.391)	<0.001	3.245 (1.731-6.082)	<0.001
Grade 5	4.977 (1.480-16.739)	0.010	4.422 (1.291-15.148)	0.018
Steroid (no vs. yes)	0.508 (0.304-0.848)	0.010		
*D*_max_ of the GTVnx (<70 vs. ≥70)	1.048 (0.583-1.885)	0.876		
*D*_max_ of the GTVnd (<64 vs. ≥64)	2.016 (1.196-3.398)	0.009	2.640 (1.513-4.607)	0.001
Radiotherapy methods (conventional vs. IMRT)	0.593 (0.355-0.991)	0.046		
Neutrophil count, ×10^9^/L (<4.47 vs. *≥*4.47)	1.840 (1.086-3.117)	0.023	2.004 (1.153-3.484)	0.014
Hemoglobin, g/L (<127.5 vs. *≥*127.5)	0.575 (0.339-0.975)	0.040		
LDL, mmol/L (<3.25 vs. ≥3.25)	0.505 (0.296-0.862)	0.012		
Prealbumin, mg/L (<0.24 vs. ≥0.24)	0.499 (0.292-0.850)	0.011		
Albumin, g/L (<39.3 vs. *≥*39.3)	0.406 (0.235-0.701)	0.001		
hsCRP, mg/L (<4.70 vs. *≥*4.70)	3.121 (1.757-5.543)	<0.001		
ESR, mm/h (<20 vs. ≥20)	6.041 (3.053-11.951)	<0.001	4.429 (2.183-8.987)	<0.001
TNM stage		0.285		
Stage 1	Reference			
Stage 2	1.452 (0.169-12.495)	0.734		
Stage 3	1.830 (0.249-13.446)	0.553		
Stage 4	2.811 (0.380-20.796)	0.312		
Secondary radiotherapy (no vs. yes)	0.668 (0.208-2.137)	0.496		
Chemotherapy (no vs. yes)	0.655 (0.310-1.383)	0.267		

## Data Availability

Data are available from the corresponding author upon reasonable request.

## Abbreviations

RAP: Radiotherapy-associated aspiration pneumonia
hsCRP: High sensitivity C-reactive protein
IMRT: Intensity-modulated radiotherapy
LDL: Low-density lipoprotein cholesterol
Hb: Hemoglobin
NPC: Nasopharyngeal carcinoma
NP: Nasopharyngeal
ESR: Erythrocyte sedimentation rate
*D*_max_ of the GTVnx: The maximum radiation dose of nasopharynx gross tumor volume
*D*_max_ of the GTVnd: The maximum radiation dose of lymph node gross tumor volume
PFS: Pneumonia-free survival
DCA: Decision curve analysis.

## Supplementary Materials

Supplementary 1 Supplementary Figure 1: flowchart. This figure showed the screening process.

Supplementary 2 Supplementary Figure 2: the Schönfeld test. Proportional hazard assumption was verified by this Schönfeld test (*P* = 0.6889). This suggested that the Cox regression model analysis can be performed.
