# Supplementary material for: Establishment and Validation of a Predictive Model for Radiation-Associated Aspiration Pneumonia in Patients with Radiation-Induced Dysphagia after Nasopharyngeal Carcinoma
Source: Behav Neurol. 2022 Aug 19;2022:6307804. doi: 10.1155/2022/6307804 (PMC9418526; doi:10.1155/2022/6307804)
Supplement: Supplementary 1 — Supplementary Figure 1: flowchart. This figure showed the screening process. [file 6307804.f1.pptx]

## Slide 1
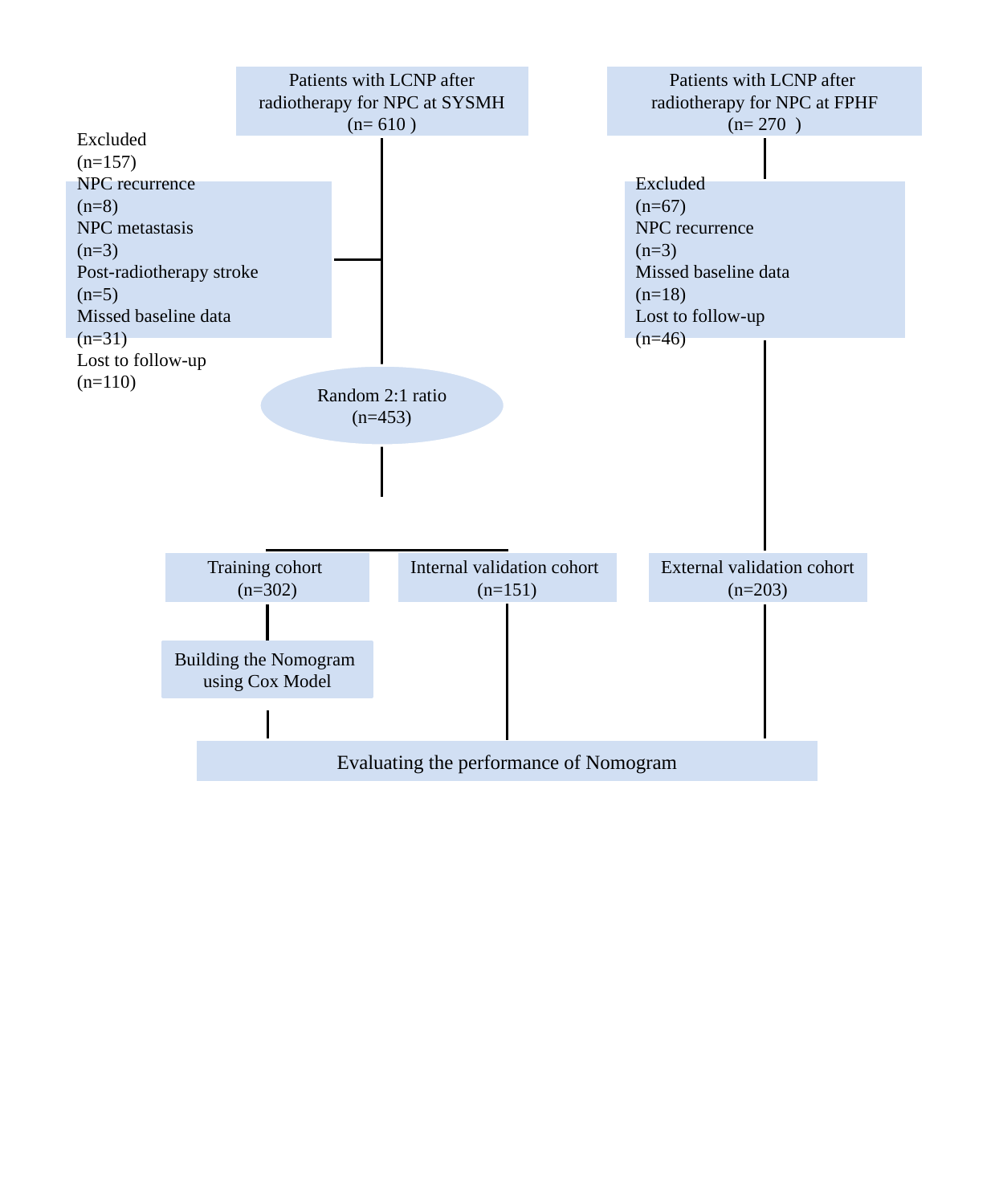

Patients with LCNP after radiotherapy for NPC at SYSMH
(n= 610 )
Patients with LCNP after
radiotherapy for NPC at FPHF
(n= 270  )
Excluded (n=157)
NPC recurrence (n=8)
NPC metastasis (n=3)
Post-radiotherapy stroke (n=5)
Missed baseline data (n=31)
Lost to follow-up (n=110)
Excluded (n=67)
NPC recurrence (n=3)
Missed baseline data (n=18)
Lost to follow-up (n=46)
Random 2:1 ratio
(n=453)
Training cohort
(n=302)
Internal validation cohort
(n=151)
External validation cohort
(n=203)
Building the Nomogram
using Cox Model
Evaluating the performance of Nomogram
