# Supplementary material for: Establishment and Validation of a Predictive Model for Radiation-Associated Aspiration Pneumonia in Patients with Radiation-Induced Dysphagia after Nasopharyngeal Carcinoma
Source: Behav Neurol. 2022 Aug 19;2022:6307804. doi: 10.1155/2022/6307804 (PMC9418526; doi:10.1155/2022/6307804)
Supplement: Supplementary 2 — Supplementary Figure 2: the Schönfeld test. Proportional hazard assumption was verified by this Schönfeld test (P = 0.6889). This suggested that the Cox regression model analysis can be performed. [file 6307804.f2.pptx]

## Slide 1
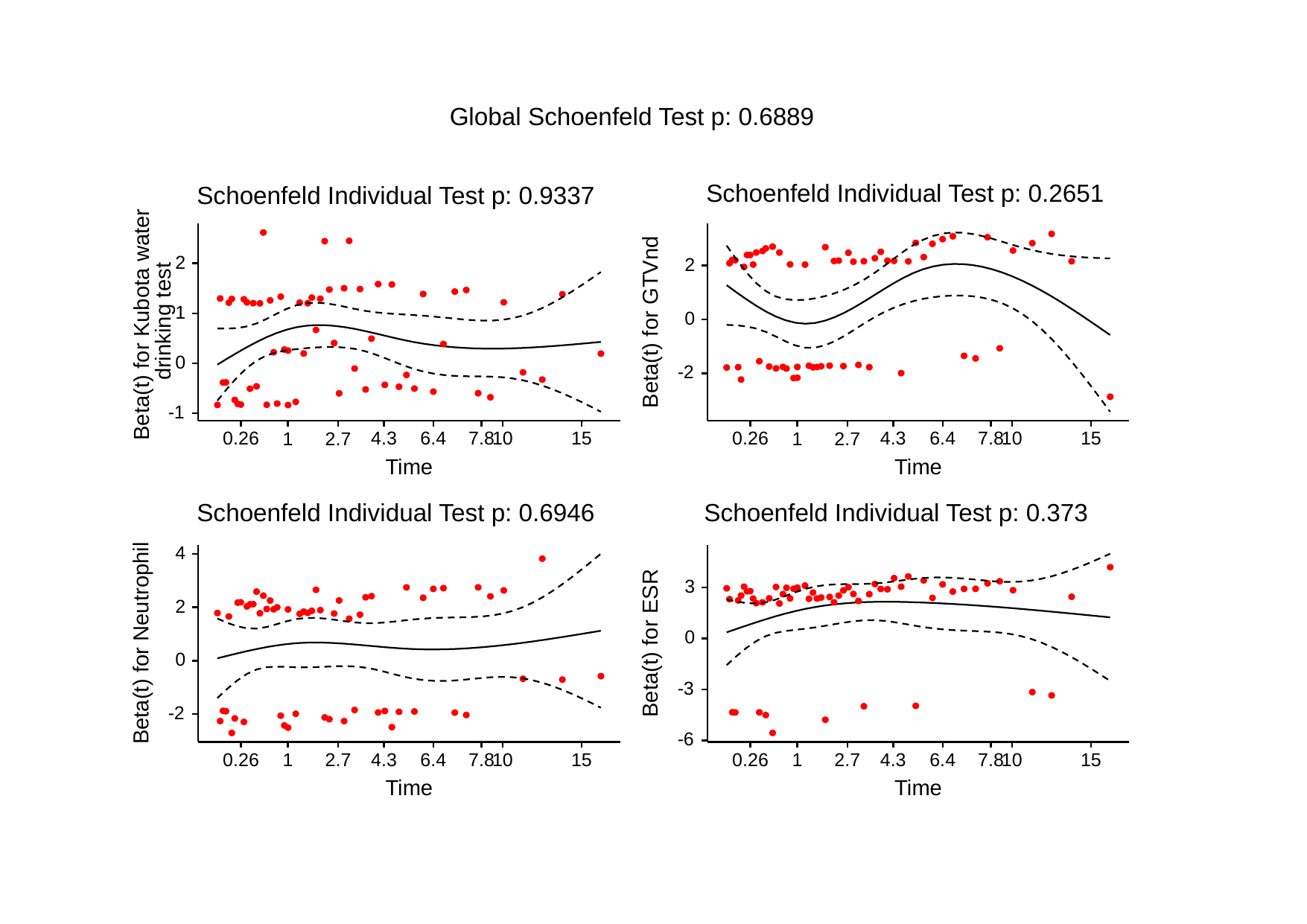

Global Schoenfeld Test p: 0.6889
Schoenfeld Individual Test p: 0.2651
Schoenfeld Individual Test p: 0.9337
2
2
Beta(t) for Kubota water
drinking test
1
Beta(t) for GTVnd
0
0
-2
-1
4.3
4.3
0.26
6.4
7.8
10
15
0.26
6.4
7.8
10
15
1
2.7
1
2.7
Time
Time
Schoenfeld Individual Test p: 0.373
Schoenfeld Individual Test p: 0.6946
4
3
2
0
Beta(t) for Neutrophil
Beta(t) for ESR
0
-3
-2
-6
4.3
4.3
0.26
6.4
7.8
10
15
0.26
6.4
7.8
10
15
1
2.7
1
2.7
Time
Time
